# Arsenic exposure through drinking water increases the risk of liver and cardiovascular diseases in the population of West Bengal, India

**DOI:** 10.1186/1471-2458-12-639

**Published:** 2012-08-10

**Authors:** Nandana Das, Somnath Paul, Debmita Chatterjee, Nilanjana Banerjee, Niladri S Majumder, Nilendu Sarma, Tanmoy J Sau, Santanu Basu, Saptarshi Banerjee, Papiya Majumder, Apurba K Bandyopadhyay, J Christopher States, Ashok K Giri

**Affiliations:** 1 Molecular and Human Genetics Division, CSIR- Indian Institute of Chemical Biology, 4, Raja S. C. Mullick Road, Kolkata, 700 032, India; 2 Sir Nil Ratan Sircar Medical College and Hospital, Kolkata, 700 014, India; 3 Department of Medicine, Calcutta National Medical College, Kolkata, 700 017, West Bengal, India; 4 Department of General Medicine, Sri Aurobindo Seva Kendra, Kolkata, 700068, West Bengal, India; 5 Regional Institute of Opthalmology, Calcutta Medical College, Kolkata, 700073, West Bengal, India; 6 Tata Main Hospital, Jamshedpur, 831001, Jharkhand, India; 7 Department of Pharmacology and Toxicology, University of Louisville, Louisville, KY, 40209, USA

**Keywords:** Arsenic, Antinuclear antibody, Liver function tests, Cytokines

## Abstract

**Background:**

Arsenic is a natural drinking water contaminant affecting 26 million people in West Bengal, India. Chronic arsenic exposure causes cancer, cardiovascular disease, liver disease, neuropathies and ocular diseases. The aims of the present study were to assess bioindicators of hepatocellular injury as indicated by the levels of liver enzymes, to determine the auto immune status, as indicated by the amounts of anti-nuclear antibodies (ANA) and anti-dsDNA antibodies in their serum, and to predict cardiovascular risk in the arsenic exposed population.

**Methods:**

Effect of chronic arsenic exposure on liver was determined by liver function tests. Autoimmune status was measured by measuring ANA and anti-dsDNA in serum. Inflammatory cytokines associated with increased cardiovascular disease risk, IL6, IL8 and MCP-1 were determined.

**Results:**

Our results indicated that serum levels of bilirubin, alanine transaminase, aspartate transaminase, alkaline phosphatase and ANA were increased in the arsenic exposed population. Serum levels of IL6 and IL8 also increased in the arsenic exposed group.

**Conclusions:**

Chronic arsenic exposure causes liver injury, increases the serum levels of autoimmune markers and imparts increased cardiovascular risk.

## Background

Arsenic is a paradoxical human carcinogen affecting millions of people around the world. At present, people in more than 35 countries across the globe are affected by drinking arsenic-contaminated ground water. In India, West Bengal is the worst affected state where more than 26 million people in 9 out of 18 districts are drinking heavily contaminated ground water through hand-pumped tube-wells [1]. The arsenic concentrations in these districts are far above the current maximum contaminant level (MCL) established by WHO and US EPA i.e. 10 μg/l [2,3]. This is regarded as the greatest arsenic calamity in the world [4]. Long term exposure to arsenic-contaminated water causes a wide range of adverse health effects, including cancer [5], cardiovascular disease [6], diabetes mellitus [7], neuropathies [8], liver disease [9], ocular diseases [8], and skin lesions [5,10]. The skin lesions include rain drop pigmentation, hypopigmentation, hyperpigmentation, keratoses and skin cancers including Bowen’s disease, basal cell carcinoma and squamous cell carcinoma [11,12]. More than 300,000 people in West Bengal have these skin lesions that are hallmarks of chronic arsenic toxicity. Prolonged arsenic ingestion leads to its accumulation in the liver, kidneys, heart and lungs and in smaller amounts in the muscles, nervous system, gastrointestinal tract and spleen [13]. Respiratory disease is also common in arsenic toxicity [8,11,14].

Among the various internal organs affected by chronic exposure to arsenic in humans, liver is one of the important targets. Epidemiological studies have shown association between chronic arsenic exposure and liver disease including hepatomegaly, hepatoportal sclerosis, liver fibrosis and cirrhosis of liver [9,15-17]. Abnormal liver functions as manifested by severe gastrointestinal problems and clinical elevations of liver enzymes in plasma including alanine amino transferase (ALT), asparatate amino transferase (AST), alkaline phosphatase (ALP) also are associated with chronic arsenic exposure [9,15,18]. Exposure of mice to arsenic in drinking water causes elevation of liver enzymes in plasma [19] and capilarization of liver sinusoidal endothelium [20]. Infact, liver is the major site of arsenic metabolism [21] and hence arsenic exposure causes liver disease in exposed humans [9].

Antinuclear antibodies (ANA) are a diverse group of autoantibodies that are found in autoimmune disorders like systemic lupus erythomatosus, Rheumatoid arthritis, Sjorgen’s syndrome, systemic sclerosis and inflammatory myositis. Arsenic exposure increases the incidence of auto-immune diseases like diabetes mellitus [7]. ANA are present in lower titers in liver diseases, leprosy, multiple sclerosis and juvenile rheumatoid arthritis [22].The association of chronic arsenic exposure and autoimmune disorders has received only minimal attention [23,24].

Inflammation has been found to be one of the most important factors that contribute to cardiovascular disease. Elevated levels of monocyte chemoattractant protein (MCP-1), interleukin 6 (IL6), and tumor necrosis factor alpha (TNFα), produced by the immune system play important roles in increasing the risk of cardiovascular disease. Both IL6 and TNFα play important roles in the regulation of the synthesis of other acute phase proteins which are established risk factors for atherosclerosis [25]. Exposure of mice to arsenic in drinking water causes the induction of inflammatory cytokines associated with increased atherosclerosis [26].

We have been studying health effects, cytogenetic damage, genetic variants, and immunological changes in the population exposed to arsenic through drinking water in West Bengal [5,10,12,27-32]. During our epidemiological survey, we found that the arsenic exposed population complains of muscular cramps and joint pains, which are characteristics symptoms of rheumatoid arthritis. In the present study we assessed bioindicators of hepatocellular injury as indicated by the serum levels of liver enzymes, determined the autoimmune status as indicated by the amounts of serum ANA and anti-dsDNA and measured circulating inflammatory cytokines related to cardiovascular diseases in the population exposed to very high arsenic content in their drinking water. The results have been compared with a group of arsenic unexposed individuals (controls). We show that the arsenic exposed population exhibits increased liver enzymes, increased serum ANA and inflammatory cytokines indicating liver injury, heightened autoimmunity, and increased likelihood of cardiovascular disease.

## Methods

### Chemicals

Ascorbic acid, acetone, nitric acid (69% GR), hydrochloric acid (35%), potassium iodide, sodium hydroxide, and sodium borohydride (96% pure) were obtained from Merck (Hohenbrunn, Germany). Arsenic (III) and arsenic (V) AAS (Atomic Absorption Spectrometry) standards were obtained from Qualigens (Shanon Co. Clare, Ireland). ELISA kits for ANA and anti-ds DNA were obtained from Calbiotech Inc. (Spring valley, CA, USA). Kits for estimation of ALT, AST,ALP, bilirubin, total protein and GGT were obtained from Randox laboratories limited (Antrim, United Kingdom). ELISA kits for IL6, IL8, and MCP-1 were obtained from Thermo Scientific (Pierce Biotechnologies, Rockford, USA).

### Study site and study population

Murshidabad district is a highly contaminated district in West Bengal India. Here people are exposed to arsenic by drinking heavily contaminated ground water, which is much above the permissible limits laid by WHO [2]. In our epidemiological survey, we initially recruited a large number of participants, from which we selected those with the highest current exposure (as measured by arsenic concentration in water and urine) in Murshidabad district for the current study. Thus, 103 arsenic exposed individuals (both with and without skin lesions) were chosen while 107 unexposed individuals were chosen from arsenic unexposed East Midnapur district, of the same state, where arsenic in drinking water ranges between 3-10 μg/L. Individuals ranging from 15 to 78 years of age with at least 10 years of exposure were included as exposed study participants. Initially, four trained volunteers were sent to the villages for door-to-door survey to identify individuals with skin lesions among the villagers. All the villagers were requested to attend the medical camp irrespective of the presence of arsenic-induced skin lesions. An interview was performed using a structured questionnaire that elicited information about demographic factors, life-style, occupation, diet, tobacco usage, medical, and residential histories [8,31]. Detailed information on current and life time tobacco usage were obtained. Of the study population 10% were randomly re-interviewed in the field to verify the accuracy of the information provided. A team of physicians consisting of specialists in the fields of dermatology, neurology, ophthalmology and respiratory diseases, each with 15 years of experience examined the study participants. Samples were collected only from those subjects who provided informed consent to participate and fulfilled the inclusion criteria. Arsenic-exposed individuals and unexposed individuals were matched with respect to age, sex, and socio-economic status. Occupationally, the majority of the study participants were farmers and household workers. In general these population are from the rural Bengal and belongs to low income group. The arsenic exposed individuals show various non cancerous, precancerous and cancerous skin lesions which are hallmarks of chronic arsenic toxicity. The non-dermatological health outcomes include conjunctivitis, peripheral neuropathy and respiratory problems [8]. In the course of our study, we have found that the arsenic-exposed individuals are more susceptible to opportunistic infections, respiratory problems, fungal and parasitic infections. Our earlier studies revealed that these individuals had impaired macrophage functions and increased death of immune cells by apoptosis [10,28].

Since arsenic containing pesticides were not very common in these areas and arsenic mining was not done in this region, occupational exposure to arsenic was ruled out. Therefore, drinking water is the principle source of arsenic in this region. Water and urine samples were collected from the subjects on the same day, which carried code numbers. Information from questionnaire-sourced data on the subjects was not revealed before arsenic analyses were completed. This study was conducted in accord with the Helsinki II Declaration (http://www.wma.net/e/policy/b3.htm) and approved by the Institutional Ethics Committee named “The Ethical Committee on Human Subjects, Indian Institute of Chemical Biology” dated April 26, 2010.

### Physical examination of skin

A careful examination of skin was conducted under natural daylight to reveal the presence of typical arsenic-induced skin lesions including raindrop, hypo- and hyper-pigmentation, palmo- and planter-hyperkeratosis. Visible or palpable dermal lesions were documented. Physical examination of skin identified three types of cancerous lesions: squamous cell carcinoma, Bowen’s disease and basal cell carcinoma. Two dermatologists with more than 15 years of experience in the relevant field, jointly diagnosed any “probable or doubtful” skin lesion. The cases that still remained doubtful were not considered. Arsenic-induced skin lesions served as a biological marker of arsenicosis. The dermatologists confirmed that the individuals without skin lesions did not develop arsenic-induced skin lesions even after prolonged arsenic exposure.

### Analysis of arsenic in water and urine samples

All the study participants were provided with acid-washed [nitric acid–water (1:1)] polypropylene bottles for collection of drinking water (approximately 100 ml) into which nitric acid (1.0 ml/L) was added later as a preservative [32]. First morning voids (approx. 100 ml) were collected in precoded polypropylene bottles for arsenic estimation as these give the best measure of the recent arsenic exposure [33]. Immediately after collection, the samples were stored in salt–ice mixture and brought to the laboratory where they were kept at –20°C until estimation of arsenic was carried out. Flow injection–hydride generation–atomic absorption spectrometry was used for the determination of total arsenic in the collected samples. A Perkin-Elmer Model-Analyst 700 (Boston, MA, USA) spectrometer equipped with a Hewlett-Packard (Houston,TX, USA) Vectra computer with GEM software, Perkin-Elmer EDL System-2, arsenic lamp (lamp current 380 mA) was utilized for the purpose [28].

### Isolation of serum from blood

Blood samples were collected by veinpuncture method. About 2 ml blood was stored aseptically in sterile 10 ml polypropylene tubes without any anti coagulant for serum collection. Care was taken to prevent any mechanical damage which might cause haemolysis of the blood. The tubes were allowed to stand in room temperature for few minutes and then put on ice and transferred to laboratory for further processing. Coagulated blood was centrifuged at 3000 rpm for 10 minutes and the clear serum was collected and stored at -80°C till analyzed.

### Assay of bilirubin, liver enzymes and autoimmune markers

Total serum protein, bilirubin and AST, ALT, ALP and GGT activities were determined in serum samples of both the exposed and unexposed individuals using a Randox Daytona autoanalyser (Randox laboratories Ltd., Crumlin, Co. Antrim, UK). Serum ANA and anti-dsDNA were determined using ELISA kit from Calbiotech Inc. (Spring valley, CA,USA) according to manufacturer’s instructions.

### Cytokine quantification

Serum levels of cytokines IL6, IL8, and MCP-1 were measured in a subset of the total population under study. A total of 65 (32 arsenic exposed and 33 arsenic unexposed) individuals were randomly chosen for measurement of serum IL6, IL8 and MCP1 levels .The individuals were matched with respect to age-sex-tobacco usage status. Serum samples were collected from coagulated blood. IL6, IL8, and MCP1 concentrations were measured by ELISA using IL6, IL8, and MCP1 ELISA kits from Thermo Scientific (Pierce Biotechnologies), following manufacturer’s instructions.

### Statistical analysis

Mann Whitney test was performed to test for significant differences in all parameters, including arsenic contents in urine, water, liver function tests, ANA, anti ds-DNA, IL6, IL8, and MCP-1 between the unexposed and exposed groups. Data are expressed as mean ± SD. One way ANOVA with Tukey-Kramar Multiple Comparisons Post-Test (for parametric data) and Kruskal-Wallis Test with Dunn’s Post test (for non parametric data) were used to compare differences in the central tendencies of different liver function and autoimmune marker parameters in more than two groups. Microsoft Excel and Graph Pad Instat (San Diego, CA, USA) software were used for the purpose.

## Results

### Demographic characteristics of study participants

Descriptive characteristics of the exposed individuals and unexposed individuals are summarized in Table 1. The average ages of unexposed and exposed individuals 40.0 ± 13.0 and 40.1 ± 13.4 years respectively. Age distribution pattern of the study participants in both the groups are shown in Table 1. Total study population is divided into six subgroups of 10 years interval. Occupationally, majority of the male individuals were farmers and females were housewives in both categories. There was no significant difference in the age or sex distribution patterns, tobacco usage and socio-economic status between the unexposed and exposed groups (Table 1).

**Table 1:** Demographic characteristic of the arsenic unexposed and exposed study participants

**Parameters**	**Unexposed individuals**	**Exposed individuals**
Total Subjects (N)	107	103
Male [N (%)]	54 (50.46)	59 (57.28)
Female [N (%)]	53 (49.53)	44 (42.71)
Age (in years, Mean ± SD)	40.03 ± 13.01	40.13 ± 13.36
Age in years (N)		
15-24	9	10
25-34	30	29
35-44	31	28
45-54	21	21
55-64	11	10
>65	5	5
Occupation [N (%)]		
Male Participants		
Farmer	49 (90.71)	50 (84.80)
Dailywage earner	0	3 (6.81)
Business	0	4 (5.12)
Teacher	2 (3.70)	0
Student	3 (5.52)	1 (1.70)
Service	0	0
Not employed	0	1 (1.70)
Female Participants		
Housewife	45 (84.92)	39 (86.63)
Student	3 (5.72)	0
Farmer	0	4 (9.12)
Not employed	1 (1.90)	1 (2.32)
Tobacco Consumption (N,%)		
Tobacco user	41 (38.30)	49 (47.64)
Tobacco non-user	66 (61.73)	54 (52.40)
Arsenic Content (Mean ± SD)		
Water (μg/L)	5.38 ± 2.06	202.68 ± 188.12*
Urine (μg/L)	18.51 ± 10.62	612.90 ± 336.91*

*p < 0.001 for for Mann Whitney test when compared to unexposed group.

### Arsenic content in urine and water samples of the study populations

Arsenic concentration was measured in water and urine samples in the study populations and the results are summarized in Table 1. Arsenic content in water of the exposed group was significantly higher than the recommended MCL of 10 μg/L established by WHO and USEPA. In the unexposed control group, the arsenic content in water was always less than 10 μg/L. Results show that the concentration of arsenic in urine of arsenic exposed individuals also was significantly higher (p < 0.001) compared to the unexposed individuals.

### Arsenic induced effects on enzyme markers of liver injury

We determined the status of liver injury in our study population by determining serum protein, bilirubin, and ALT, AST, ALP and GGT activities (Table 2). Bilirubin, AST, ALT, and ALP were increased in the exposed individuals, indicating chronic arsenic exposure had caused liver injury and malfunction in these people.

**Table 2:** Serum levels of the liver function test parameters in arsenic exposed and unexposed population

**Parameters**	**Unexposed individuals**	**Exposed Individuals**
Total Subjects (N)	107	103
Bilirubin (mg/dl)	0.59 ± 0.21	0.70 ± 0.27*
AST (U/L)	44.01 ± 9.06	51.72 ± 11.34**
ALT (U/L)	31.80 ± 9.41	36.60 ± 11.07***
Total Protein (g/dl)	7.94 ± 1.29	8.30 ± 0.78
ALP (U/L)	216.69 ± 57.29	274.03 ± 84.62****
GGTP (U/L)	13.14 ± 3.92	14.12 ± 8.19

*p = 0.0137 for Mann Whitney Test when compared to unexposed group.

**p < 0.0001 for Students Unpaired Test when compared to unexposed group.

***p = 0.0012 for Mann Whitney Test when compared to unexposed group.

****p < 0.0001 for Mann Whitney Test when compared to unexposed group.

### Arsenic induced effects on autoimmunity markers

The arsenic exposed population very often complains of muscular cramps and joint pain, which are typical symptoms of rheumatoid arthritis (an autoimmune disorder). Therefore, we predicted that autoimmunity markers might be elevated in the arsenic exposed population. Thus, we determined the serum levels of ANA and anti-dsDNA in our study population. We found that ANA levels were increased in the arsenic exposed population (Table 3). Interestingly, we did not find any difference in serum levels of anti-dsDNA in the unexposed and exposed groups.

**Table 3:** Serum levels of ANA and Anti-dsDNA in the arsenic exposed and unexposed population

**Parameters**	**Unexposed individuals**	**Exposed Individuals**
Total Subjects (N)	107	103
ANA^a^	0.94 ± 0.53	1.20 ± 0.82*
Anti ds DNA	0.71 ± 0.29	0.72 ± 0.48

*p < 0.05 for Mann Whitney Test when compared to unexposed group.

### Dose response relationship with urine arsenic concentration

We have grouped our exposed study population into four sub-groups depending upon the arsenic concentration in their urine. Group A consists of individuals having arsenic content in urine below 300 ppb, group B, C and D consists of individuals having urinary arsenic concentration of 301-600 ppb, 601-900 ppb and more than 900 ppb respectively. The results are shown in Table 4 which indicates that there is a significant increase in both liver function test parameters (AST and ALT) and autoimmune markers (ANA and anti-dsDNA) with increase in arsenic content in urine.

**Table 4:** Dose response measurement of liver function test parameters and autoimmune markers with urinary arsenic concentration

	**Group A (N = 15)**	**Group B (N = 40)**	**Group C (N = 24)**	**Group D (N = 24)**	**P –Values for dose response effects**
Bilirubin (mg/dl)	0.63 ± 0.28	0.68 ± 0.23	0.71 ± 0.33	0.69 ± 0.21	0.872
AST (U/L)	48.70 ±16.35	51.65 ± 8.47	57.86 ± 10.61	51.92 ± 8.43	0.039*
ALT (U/L)	30.87 ± 7.87	37.95 ± 9.26	40.58 ± 9.91	41.38 ± 12.88	0.013*
Total Protein (g/dl)	8.18 ± 0.77	8.19 ± 0.78	8.44 ± 0.85	8.41 ± 0.72	0.564
ALP (U/L)	216.87 ± 86.49	221.41 ± 50.74	215.25 ± 133.65	241.67 ± 67.27	0.098
GGTP (U/L)	11.67 ± 2.16	13.27 ± 6.65	14.25 ± 9.08	16.91 ± 11.12	0.172
ANA	0.85 ± 0.34	1.24 ± 1.10	1.18 ± 0.62	1.35 ± 0.63	0.025**
Anti ds DNA	0.65 ± 0.33	0.77 ± 0.50	0.53 ± 0.37	0.88 ± 0.60	0.017**

Group A = Urine arsenic concentration 0-300 ppb.

Group B = Urine arsenic concentration 301-600 ppb.

Group C = Urine arsenic concentration 601-900 ppb.

Group D = Urine arsenic concentration >900 ppb.

* ANOVA with Tukey-Kramer Multiple Comparisons Test.

** Kruskal-Wallis Test with Dunn’s post test.

### Arsenic induced secretion of cardiovascular markers

IL6, IL8 and MCP-1 are inflammatory cytokines associated with cardiovascular disease. Thus, we determined the circulating levels of these cytokines as indicators of cardiovascular disease associated with arsenic exposure (Table 5). We have found that IL6 and IL8 were increased significantly in the exposed group. The apparent increase in MCP-1 was not statistically significant.

**Table 5:** Comparison of three cytokine levels in serum of arsenic exposed and unexposed individuals

**Cytokines (pg/ml)**	**Unexposed individuals**	**Exposed Individuals**
Total Subjects (N)	33	32
IL6	4.11 ± 1.98	5.63 ± 2.92*
IL8	14.00 ± 8.54	38.11 ± 17.27**
MCP-1	106.42 ± 62.07	130.68 ± 86.91

*p = 0.018 for Mann Whitney Test when compared to unexposed group.

**p < 0.001 for Mann Whitney test when compared to the unexposed group.

## Discussion

Chronic arsenic exposure is well established as carcinogenic but interest in the non-cancer disease endpoints of arsenic exposure is of great interest. The non-cancer disease endpoints include cardiovascular disease [6] and immune dysfunction [34], as well as neuropathies and ocular diseases [8,35,36]. In the present study, we focused on biological markers of liver disease, autoimmunity and cardiovascular disease in a population in West Bengal exposed to high levels of arsenic in drinking water. Increased bilirubin and liver enzyme levels in serum indicated the presence of liver injury in the arsenic exposed individuals. Increased serum ANA indicated increased likelihood of autoimmunity, and increased serum inflammatory cytokine levels indicated increased systemic inflammation and risk of cardiovascular diseases in the arsenic exposed individuals.

Liver function tests are a helpful screening tool to detect hepatic dysfunction. Liver has to perform different kinds of biochemical, synthetic and excretory functions, so no single biochemical test can detect the global functions of liver. To detect the proper functioning of the liver, various tests are performed to detect specific liver activities. Among various parameters, serum bilirubin is the marker the liver’s capacity to transport organic anions and to metabolize drugs. Aminotransferases (ALT, AST), alkaline phosphatase (ALP), aminopeptidase are the tests to detect injury to hepatocytes. Total protein is the marker for liver’s biosynthetic capacity [37]. Bilirubin is an endogenous anion derived from hemoglobin degradation. Underlying liver disease is indicated when the liver function tests are abnormal and serum bilirubin levels are elevated. Hyperbilirubinemia seen in acute viral hepatitis is directly proportional to the degree of histological injury of hepatocytes and the longer course of the disease [38]. The aminotransferases, AST and ALT are the most frequently utilized indicators of hepatocellular necrosis. ALT is primarily localized to the liver but the AST is present in a wide variety of tissues like the heart, skeletal muscle, kidney, and brain as well as liver. The AST and ALT levels are increased to some extent in almost all liver diseases. The highest elevations occur in severe viral hepatitis, drug or toxin induced hepatic necrosis and circulatory shock [38]. Higher levels of alkaline phosphatase occur in cholestatic disorders. Elevations occur as a result of both intrahepatic and extrahepatic obstruction to bile flow and the degree of elevation does not help to distinguish between the two. The mechanism by which alkaline phosphatase reaches the circulation may be due to leakage from the bile canaliculi into hepatic sinusoids via leaky tight junctions [39]. In healthy people most circulating alkaline phosphatase originates from liver or bone [40]. In liver disease, glutamyl transpeptidase activity correlates well with alkaline phosphatase levels [41]. So, clinicians are often confused when they see elevated alkaline phosphatase levels and are unable to differentiate between liver diseases and bony disorders and in such situations measurement of glutamyl transferase helps as it is raised only in cholestatic disorders and not in bone diseases. Elevated levels of these liver function enzymes have been associated with hepatic dysfunctions in lead [42,43] and cadmium [44] exposure. In our study, we have found that serum levels of bilirubin, ALT, AST, and ALP have increased significantly in the arsenic exposed population when compared to the unexposed group with similar socio-economic status. These increases clearly indicate that chronic arsenic exposure causes injury to hepatocytes with damage to the liver’s capacity to transport organic anions and to metabolize drugs, cholestatic injury and impairment of the liver’s biosynthetic capacity. Our results are consistent with many findings of chronic exposure to arsenic associated with hepatomelagy, hepatoportal sclerosis, liver fibrosis and cirrhosis with concomitant increase in serum bilirubin, ALT, AST, and ALP [9,45,46].

ANA and anti-ds DNA are frequently found in serum of patients with different types of autoimmune disorders and are biomarkers of autoimmune disorders [47-50]. Rheumatic diseases that affect connective tissue, including the joints, bone, and muscle are associated with these antibodies. Autoimmune and rheumatic diseases can be difficult to diagnose. People with the same disease can have very different symptoms. A helpful strategy in the diagnosis of these diseases is to find and identify an autoantibody in the person's blood. ANA bind to several nuclear antigens. It is useful as a screen for many autoantibodies associated with systemic diseases. Presence of anti-dsDNA is one of the diagnostic criteria for systemic lupus erythomatosus, which is an autoimmune disorder. IgG class antibodies, antibodies to single stranded DNA (ssDNA) and IgM antibodies to DNA are found in number of connective diseases as well as in rheumatoid disorders [51,52]. Increased ANA titres have also been reported in arsenic exposed population earlier [23]. Thus, ANA can be utilized as a biomarker in early risk assessment of arsenic induced autoimmune disease in high risk arsenic zones, since most of the individuals did not show any symptoms of rheumatoid arthritis although they were ANA positive. We have also found a significant increase in the serum levels of ANA and anti ds DNA in arsenic exposed population compared to the unexposed population in West Bengal. Therefore ANA appears to be a useful biomarker in early risk assessment of arsenic exposure induced autoimmune disease, which might lead to rheumatoid arthritis or other types of autoimmune diseases.

In order to find out the dose response of the LFT parameters and autoimmune markers with the increase of arsenic concentration in urine, we have stratified the data of the exposed population into four sub-groups depending upon the arsenic content in urine as mentioned in the results section. Our findings show that an increasing trend is observed in both LFT parameters and autoimmune markers with increasing urinary arsenic concentration. The dose response increase is significant in case of AST, ALT, ANA and anti-dsDNA indicating that with increase in arsenic exposure (as urine is the best current exposure marker), the levels of hepatic damage and autoimmune response increases in the exposed individuals. Although no dose response is observed in bilirubin and alkaline phosphatase levels with urinary arsenic content compared to unexposed group , the significant increase in levels of other hepatic parameter indicates that increase in arsenic exposure causes hepatic injury since all the parameters together contribute to hepatic damage.

Cytokines play pivotal roles in systemic inflammation and thus in cardiovascular diseases [52]. In atherosclerotic plaque, inflammatory cytokines (IL6, IL8, MCP-1, TNFα, IL1b) secreted by macrophages, dendritic cells, T cells and smooth muscle cells, aggravate plaque instability by inhibiting extracellular matrix synthesis and promoting smooth muscle cell apoptosis. Elevated levels of IL8 found in atherosclerotic plaques suggest that it acts as important mediator of angiogenesis in this tissue contributing to plaque formation [53]. Elevated plasma levels of IL6 and TNFα were detected in patients with stable or unstable angina and myocardial infarction [54,55]. It was also found that increased plasma levels of IL6, IL8 and MCP-1 were associated with cardiovascular risk in patients with systemic lupus erythematosus [56]. Arsenic exposure has been connected with a host of cardiovascular risk factors and endpoints including peripheral arterial disease (blackfoot disease), atherosclerosis, coronary heart disease, stroke, and hypertension [57]. It has been found that the expression of several cytokine related genes is increased in human subjects with increase in arsenic exposure, including heme oxygenase-1 (HO-1), MCP-1 and IL6 [58]. The secretion of theses cytokines are regulated by reactive oxygen species (ROS) and they are involved in modulating the biological functions of vascular smooth muscle cells are thus are involved in atherosclerosis [59]. In our previous study we have found increased generation of ROS [10] in the arsenic exposed population of West Bengal. The increased oxidative stress due to arsenic exposure may influence inflammatory responses in the vascular cells. We can conclude that increased serum concentrations of these cytokines and chemokines (IL6, IL8 and MCP-1) may act as early biomarkers of increased cardiovascular risk in the arsenic exposed subjects. In this context it is worthwhile to discuss that Kupffer cells in the liver are a major source of the inflammatory cytokines upon hepatotoxic insult [60]. Our results show that liver injury by arsenic exposure. Thus, the injured hepatic cells may induce increased secretion of the inflammatory cytokines (IL6, IL8, TNFα) in the arsenic exposed individuals which in turn contributes to cardiovascular risk.

## Conclusions

Chronic arsenic exposure causes a wide variety of human diseases. Exposed individuals are at higher risk of developing liver and cardiovascular disease, as indicated by elevated serum levels of liver injury biomarkers and inflammatory cytokines. Increase of autoimmune markers in the serum suggests that arsenic exposure also induces autoimmune diseases such as rheumatoid arthritis. Both rheumatoid arthritis and liver disease are risk factors for cardiovascular disease. Thus, the arsenic exposure induced systemic inflammatory disease and the hepatic injury likely contributes to increased cardiovascular risk observed in arsenic exposed populations.

## Abbreviations

ALP, Alkaline phosphatase; ALT, Alanine transaminase; ANA, Anti-nuclear antibody; AST, Aspartate transaminase; GGT, γ-Glutamyl transpeptidase; IL6, Interleukin 6; IL8, Interleukin 8; MCL, Maximum contaminant level; MCP-1, Monocyte chemoattractant protein-1; TNFα, Tumor necrosis factor alpha.

## Competing interests

The authors declare that there exists no competing financial interest among any of the authors.

## Authors’ contributions

AKG and JCS planned, designed and wrote the final manuscript, ND and SP collected samples, performed experimental works and prepared manuscript draft, NB performed experimental works and wrote manuscript draft, DC and NSM collected samples and performed field works, NS, TJS, SB, PM, AKB performed epidemiological survey. All authors read and approved the final manuscript.

## Authors’ information

Dr. Ashok K. Giri is a Chief Scientist at the CSIR-Indian Institute of Chemical Biology. His work includes various epidemiological and molecular biological aspects of the highly arsenic exposed population of West Bengal. He has more than 20 peer reviewed publications projecting research work of more than 10 years in this area.

## Pre-publication history

The pre-publication history for this paper can be accessed here:

http://www.biomedcentral.com/1471-2458/12/639/prepub
